# ANLN is a prognostic biomarker independent of Ki-67 and essential for cell cycle progression in primary breast cancer

**DOI:** 10.1186/s12885-016-2923-8

**Published:** 2016-11-18

**Authors:** Kristina Magnusson, Gabriela Gremel, Lisa Rydén, Victor Pontén, Mathias Uhlén, Anna Dimberg, Karin Jirström, Fredrik Pontén

**Affiliations:** 1 Department of Immunology, Genetics and Pathology, Science for Life Laboratory, Uppsala University, Uppsala, Sweden; 2 Department of Clinical Sciences, Division of Surgery, Lund University, Lund, Sweden; 3 Science for Life Laboratory, KTH – Royal Institute of Technology, Stockholm, Sweden; 4 Department of Clinical Sciences, Division of Oncology and Pathology, Lund University, Lund, Sweden

**Keywords:** ANLN, Prognostic biomarker, Breast cancer, Proliferation, Antibody-based proteomics

## Abstract

**Background:**

Anillin (ANLN), an actin-binding protein required for cytokinesis, has recently been presented as part of a prognostic marker panel in breast cancer. The objective of the current study was to further explore the prognostic and functional value of ANLN as a single biomarker in breast cancer.

**Methods:**

Immunohistochemical assessment of ANLN protein expression was performed in two well characterized breast cancer cohorts (*n* = 484) with long-term clinical follow-up data and the results were further validated at the mRNA level in a publicly available transcriptomics dataset. The functional relevance of ANLN was investigated in two breast cancer cell lines using RNA interference.

**Results:**

High nuclear fraction of ANLN in breast tumor cells was significantly associated with large tumor size, high histological grade, high proliferation rate, hormone receptor negative tumors and poor prognosis in both examined cohorts. Multivariable analysis showed that the association between ANLN and survival was significantly independent of age in cohort I and significantly independent of proliferation, as assessed by Ki-67 expression in tumor cells, age, tumor size, ER and PR status, HER2 status and nodal status in cohort II. Analysis of ANLN mRNA expression confirmed that high expression of ANLN was significantly correlated to poor overall survival in breast cancer patients. Consistent with the role of ANLN during cytokinesis, transient knock-down of ANLN protein expression in breast cancer cell lines resulted in an increase of senescent cells and an accumulation of cells in the G2/M phase of the cell cycle with altered cell morphology including large, poly-nucleated cells. Moreover, ANLN siRNA knockdown also resulted in decreased expression of cyclins D1, A2 and B1.

**Conclusions:**

ANLN expression in breast cancer cells plays an important role during cell division and a high fraction of nuclear ANLN expression in tumor cells is correlated to poor prognosis in breast cancer patients, independent of Ki-67, tumor size, hormone receptor status, HER2 status, nodal status and age.

**Electronic supplementary material:**

The online version of this article (doi:10.1186/s12885-016-2923-8) contains supplementary material, which is available to authorized users.

## Background

Breast cancer is the most common female malignancy world-wide and approximately 500 000 women succumb to the disease annually [1]. In Sweden, approximately 9 100 cases of female malignant breast tumors are diagnosed annually. The incidence of breast cancer has shown an annual increase with 1.4% during the last 20 years, at least in part due to an ageing population with increased hormonal replacement therapy and changes in life style, such as obesity and first pregnancy late in life. Furthermore, systematic mammographic screening programs and elevated public awareness have led to the detection of more cases of breast cancer at an early stage. Early detection and a transition to more individualized targeted therapies, has resulted in increased recurrence-free and overall survival rates [2]. Although prognostic gene expression-based profiles have rapidly evolved, there is a need for robust immunohistochemistry (IHC)-based protein biomarkers that can be introduced into clinical praxis.

The actin-binding protein ANLN is a ubiquitously expressed protein required for cytokinesis. During the interphase of the cell cycle ANLN is primarily located to the nucleus. At the onset of mitosis, ANLN protein relocates to the cytoplasm where it accumulates in the contractile ring and cleavage furrow during telophase [3]. Recruitment of ANLN to the cleavage furrow is mediated by RhoA-dependent mechanisms [4, 5]. Furthermore, ANLN interacts closely with RhoA, stabilizes the localization of the latter to the cleavage furrow and stimulates the expression of active RhoA [4, 6]. Numerous additional proteins, including F-actin, myosin, septins and CD2AP have been shown to interact with ANLN during assembly, maintenance and ingression of the cleavage furrow [7]. Lack of ANLN is generally associated with correct assembly of the cleavage furrow but deficiencies during furrow ingression and completion of cell separation [3, 5].

Consistent with the prominent role of ANLN during cytokinesis, up-regulation of ANLN expression is frequently observed during cancer development, growth and progression [8–10]. It has also been shown that depletion of ANLN expression in human non-small cell lung cancer cells leads to suppression of cell proliferation and an increase of large, poly-nucleated tumor cells [6]. Interestingly, overexpression of the ANLN protein did not only induce cell growth, but also enhanced the migratory capacity of cells, implying a role of ANLN beyond cell cycle control. High ANLN mRNA expression and nuclear ANLN protein expression in lung cancer tissue has been shown to be significantly correlated to poor survival [6, 11]. In another study, cytoplasmic immunoreactivity for ANLN in renal cell carcinomas was associated with a better prognosis, indicating an independent function of ANLN in the cytoplasm [12]. Moreover, ANLN mRNA expression was shown to increase from normal tissue to hyperplasia to malignant and metastatic disease in breast, ovary, renal, colorectal, hepatic, lung, endometrial and pancreatic cancer [8].

The relevance of ANLN protein expression in breast cancer tissue specimens has been explored as a part of a systematic approach to identify novel prognostic biomarkers. O’Leary and co-workers [13] found that a moderate to strong nuclear intensity of ANLN expression was significantly associated with decreased breast cancer specific survival (BCSS) and recurrence free survival (RFS). Using multivariable cox regression analysis, ANLN was suggested as an independent prognostic factor for BCSS following adjustment for tumor size, nodal status, tumor grade, hormone receptor status, HER2 status, Ki-67, tumor type, age and the proteins PDZ-Domain Containing 1 (PDZK1) and PDZ-Binding Kinase (PBK). In a recent study based on a cohort consisting of 71 patients diagnosed with primary breast cancer, the rate of ANLN expression was shown to be significantly higher in breast cancer compared to normal breast tissue [14]. In this study, ANLN knockdown was also shown to inhibit cell migration, colony formation and cell cycle progression.

The aim of the present study was to further investigate and validate the prognostic significance of ANLN expression in breast cancer. Moreover, the functional role and a potential treatment predictive value of ANLN expression in patients with primary breast cancer were explored.

## Methods

### Patient cohorts

Tissue microarray (TMA) construction, IHC and slide scanning were performed as previously described [15]. TMAs with tumor samples from two independent breast cancer cohorts were used to investigate the expression of ANLN protein by IHC. All formalin-fixed and paraffin-embedded (FFPE) patient tissue samples were histopathologically re-evaluated on hematoxylin and eosin stained slides prior to TMA construction. Cohort I consisted of 144 patients diagnosed with breast cancer at Malmö University Hospital, Malmö, Sweden, between 2001 and 2002 [16, 17]. The median age at diagnosis was 65 years (range 34-97) and the median follow-up time for disease specific and overall survival was 78 months. The second cohort was comprised of 564 premenopausal breast cancer patients enrolled in a randomized tamoxifen treatment trial [18–21]. Between the years 1986 and 1991, premenopausal women with stage II breast cancer were randomized to either 2 years of tamoxifen treatment (*n* = 276) or no adjuvant treatment (*n* = 288) irrespective of hormone receptor status. The median age at diagnosis, in both treatment groups, was 45 years (range 26–57 for the control group and range 25–57 for the tamoxifen group). The median follow-up time for patients without a breast cancer event was 13.9 years. This study was approved by the local Ethics Committees at Lund and Linköping Universities, whereby informed consent was deemed not to be required but opting out was an option (cohort I) and oral informed consent (cohort II) was registered for included patients.

### Immunohistochemistry

The specific target binding of the primary affinity purified polyclonal antibody towards ANLN (HPA005680, Atlas Antibodies, Stockholm, Sweden) was initially validated according to standardized procedures used in the Human Protein Atlas (http://www.proteinatlas.org) with assays including reverse phase protein array, Western blot, IHC, immunofluorescence (IF) and comparing results with bioinformatic predictions and published data [22]. Moreover, this polyclonal ANLN antibody was further validated by epitope mapping [23].

For IHC analysis of protein expression, TMA blocks were cut in 4 μm sections using a microtome, Microm HM355S, with a section transfer system (Thermo Fisher Scientific, Waltham, USA) and placed onto Superfrost Plus glass slides and dried at room temperature over night. Following that, the slides were baked at 50°C for 12–24 h. Sections were deparaffinized in Neo-Clear (Merck, Darmstadt, Germany), hydrated in graded ethanol and blocked for endogenous peroxidase activity with 0.3% hydrogen peroxidase (Merck) in an Autostainer XL (Leica Microsystems, Wetzlar, Germany). Heat induced antigen retrieval was done by boiling the glass slides in citrate buffer, pH 6.0 (Thermo Fisher Scientific) for 4 min at 125°C in a decloaking chamber (Biocare Medical, CA, USA). Automated IHC was done as described previously [15] using a Lab Vision Autostainer 480 (Thermo Fisher Scientific). ANLN antibody (Atlas Antibodies) was diluted (1:50) in UltraAb Diluent (Thermo Fisher Scientific) and incubated on the slides for 30 min at room temperature. Following incubation with a secondary anti-rabbit antibody conjugated to a horseradish peroxidase labeled polymer (Thermo Fisher Scientific) for 30 min at room temperature, the signal was developed with diaminobenzidine (DAB) mixed with chromogen (Thermo Fisher Scientific) at 1:40 for 10 min at room temperature. Counterstaining, dehydration and mounting were done in an Autostainer XL (Leica Microsystems). Counterstaining was done with Mayer’s hematoxylin (Histolab, Gothenburg, Sweden) for 5 min at room temperature. The slides were washed in water, incubated in lithium carbonate for 1 min at room temperature, washed in water and dehydrated in graded ethanol and Neo-Clear (Merck) followed by automated coverslipping (CV5030, Leica) with Pertex (Histolab).

### Scanning and annotation

**Fig. 1:**
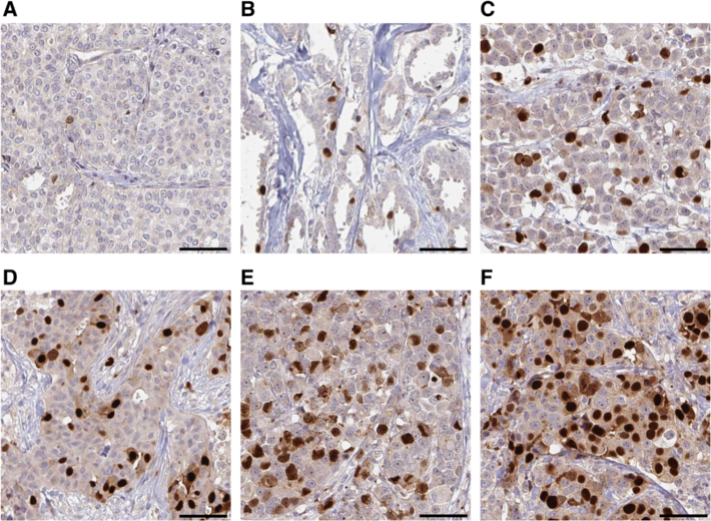
ANLN expression in breast cancer. Examples of immunohistochemical staining patterns of ANLN in breast cancer tissue shows nuclear expression in a variable fraction of tumor cells. Examples correspond to the different scores for nuclear fraction (NF) used in the analysis. 0–1% (**a**), 2–10% (**b**), 11–25% (**c**), 26–50% (**d**), 51–75% (**e**) and 76–100% (**f**). Scale bars 100 μm

The automated scanning system ScanScope XT (Aperio Technologies, Vista, USA) was used to digitalize IHC stained slides at 20x magnification. The outcome of immunohistochemical staining was manually annotated by KM, assisted by two pathologists (FP and KJ), using the Aperio ImageScope Viewer v.10.2.1.2314 (Aperio Technologies). Nuclear staining of ANLN in tumor cells was assessed from both cores (1 mm diameter) by scanning through the tumor tissue at high power magnification to estimate the fraction and intensity of positive tumor cell nuclei. The fraction of positive nuclei (NF) was categorized as 0–1%, 2–10%, 11–25%, 26–50%, 51–75% or 76–100%, and the nuclear intensity (NI) was recorded using a 4-graded scale as negative, weak, moderate or strong. Immunohistochemical staining for ANLN expression showing different levels of ANLN expression is demonstrated in Fig. 1. High ANLN expression was considered as NF > 10% independent of nuclear staining intensity.

### Cell culture

The functional relevance of ANLN was studied in two different breast cancer cell lines, one cell line that lacks ER expression (SKBR3) and one ER expressing cell line (T47D) (American Type Culture Collection, Manassas, USA). SKBR3 cells were grown in McCoy’s 5A medium (Sigma-Aldrich, St. Louis, USA) and T47D cells were grown in RPMI medium 1640 (Sigma-Aldrich), both supplemented with 10% fetal bovine serum (FBS, Invitrogen, Carlsbad, USA), 2 mM L-glutamine (Invitrogen), 50 IU/ml penicillin and 50 μg/ml streptomycin sulphate (Invitrogen). Cells were maintained in 5% CO_2_ at 37°C in a cell culture incubator (Sanyo Electric Co, Osaka, Japan). The cell lines were confirmed to be free of Mycoplasma contamination (MycoAlert Mycoplasma Detection Kit, Lonza, Rockland, USA).

### siRNA mediated gene knockdown

Two Silencer Select siRNAs targeting ANLN, s28983 (siRNA 1) and s28984 (siRNA 2) (Ambion, Applied Biosystems, Foster City, USA) were used to deplete the expression of ANLN in SKBR3 and T47D cells. A non-targeting siRNA, s229174, (Ambion) was used as control. Cells were seeded in antibiotic-free medium into six-well plates or eight-well glass chamber slides 24 h prior to siRNA transfection that was done as instructed by the manufacturer using Lipofectamine RNAiMAX (Invitrogen) as transfection reagent.

### Statistical analysis of transcriptomics data

The publically available Cancer Genome Atlas database (http://cancergenome.nih.gov/), including human transcriptomics data based on mRNA sequencing (RNA-Seq) was used to extract data for 664 patients with invasive breast cancer and clinical survival data. A log rank test was used to analyze the correlation between ANLN expression and patient survival. Tumor samples were stratified into two groups using the median Reads Per Kilobase of transcript per Million mapped reads (RPKM) value for ANLN as cut-off for a Kaplan-Meier estimate.

### Cell cycle analysis

Growth medium and subconfluent cells were collected, washed in PBS and fixed in ice-cold 70% ethanol at 4°C over night. Cells were then washed twice in ice-cold PBS, stained with 20 μg/ml propidium iodide (PI, Sigma-Aldrich) in PBS, supplemented with 60 μg/ml RNAse A (Sigma-Aldrich), for 30 min at room temperature and analyzed with a BD LSR II multi-laser analytical flow cytometer (BD Biosciences). Cell cycle data was analyzed by ModFit LT 3.2 software (Verity Software House, Topsham, USA).

### Immunofluorescence

Following siRNA transfection in eight-well glass chambers slides (BD Biosciences, Bedford, USA), cells were fixed in 4% paraformaldehyde for ten minutes at room temperature, permeabilized with 0.2% Triton X-100 for 20 min at room temperature and blocked in 5% normal goat serum for one hour at room temperature. The primary ANLN antibody (Atlas Antibodies) was added (dilution 1:100) and cell slides incubated at 4°C over night. For signal detection, a secondary anti-rabbit antibody conjugated to fluorescein isothiocyanate (FITC) (Jackson ImmunoResearch, West Grove, USA) was added and incubated for 1 h at room temperature. Actin filaments were stained with Phalloidin-Tetramethylrhodamine (TRITC) (Sigma-Aldrich) for 40 min at room temperature and the slides mounted with 4′,6-Diamidino-2-phenylindole (DAPI)-containing mounting medium (Thermo Fisher Scientific). All images were acquired with a Zeiss 510 confocal microscope using the 40X objective (Zeiss, Oberkochen, Germany).

### Senescence assay

Cellular senescence was detected using a commercially available senescence β-galactosidase staining kit (Cell Signaling Technology, Danvers, USA), according to the manufacturer’s guidelines. Briefly, fixative solution was applied for ten minutes at room temperature, the cells rinsed with PBS and incubated in β-Galactosidase Staining Solution at 37°C for approximately 12 h. The number of senescent cells was counted in three separate fields at 20x magnification. All images were acquired with a Nikon camera using the 20X objective and Infinity analyze 6.2.0 software (Lumenera, Ottawa, Canada).

### Western blot

Total cellular protein was extracted with radio-immunoprecipitation assay (RIPA) buffer (Sigma-Aldrich) supplemented with protease inhibitors (Sigma-Aldrich) 72 and 120 h after siRNA-transfection. Protein concentration was estimated with a Bicinchoninic Acid (BCA) Kit for Protein Determination (Sigma-Aldrich). Protein lysates were separated on 4–20% Criterion TGX Precast SDS-PAGE Gels (Bio-Rad Laboratories, Hercules, USA) and blotted onto PVDF membranes (Bio-Rad). Membranes were blocked with 5% milk in Tris-buffered saline containing 0.5% Tween-20 for 1 h followed by primary antibody incubation at 4°C over night. Membranes were incubated with species specific horse radish peroxidase (HRP)-conjugated secondary antibodies (DAKO) at room temperature for 1 h and developed with Immobilon Western Chemiluminescent HRP Substrate (Millipore, Billerica USA). Chemiluminescence was detected using a CCD-camera (Bio-Rad).

### Statistical analysis

Spearman’s correlation test was used to evaluate correlation between ANLN NF and NI. Differences in distribution between ANLN expression and clinicopathological parameters were evaluated by means of the Chi-square test and Fisher’s exact test for categorical and categorized variables and for ordinal variables with more than two categories a linear-by-linear test for association was used. The Kaplan-Meier method and log-rank test was used to illustrate differences in survival according to ANLN mRNA and protein expression. The Cox regression proportional hazards model was used to estimate the impact of ANLN on overall survival (OS), BCSS and RFS in univariable and multivariable analysis. The student *t*-test was used to determine the significance of functional differences between various experimental conditions during in vitro experiments. All in vitro data represents mean values derived from at least three independent experiments. All statistical tests were two-sided and *p*-values <0.05 considered significant. All calculations were performed with Microsoft Office Excel 2007 (Microsoft, Redmond, USA) or IBM SPSS Statistics version 22.0 (SPSS Inc. Illinois, USA).

## Results

### Nuclear fraction of ANLN expression is significantly associated with clinicopathological parameters

**Fig. 2:**
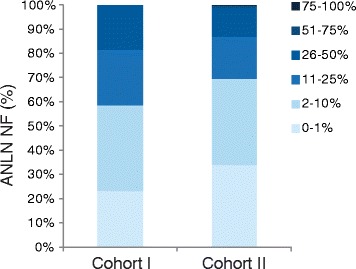
Distribution of ANLN nuclear fraction. Distribution of ANLN nuclear fraction was analyzed in two independent breast cancer cohorts. The majority of tumors investigated expressed less than 25% of ANLN nuclear staining

To validate the relevance of ANLN as a prognostic marker in breast cancer, ANLN protein expression was analyzed in two independent TMA cohorts comprising of 144 (Cohort I) and 564 (Cohort II) patients, respectively. As a consequence of missing representative tumor tissue, the ANLN expression status was evaluated in 126/144 (87.5%) tumors in cohort I and in 358/564 (63.5%) tumors in cohort II. Using Spearman’s correlation test, we found that ANLN NF was significantly associated with ANLN NI (Cohort I: correlation coefficient = 0.435, *p* < 0.001. Cohort II: correlation coefficient = 0.537, *p* < 0.001). A high percentage of primary tumors (95.2% in cohort I and 82.1% in cohort II) displayed moderate to strong nuclear intensity. The fraction of ANLN positive tumor cells varied but the majority of cases showed a NF < 25% (Fig. 2).

We investigated the potential association between ANLN NF and various clinicopathological parameters by chi-square test, linear-by-linear test or Fisher’s exact test and found that tumors with a high ANLN NF were significantly associated with an older age at diagnosis (Cohort I: *p* = 0.043), a larger tumor size (Cohort I: *p* = 0.004. Cohort II: *p* = 0.021), hormone receptor negativity (Cohort I: ER *p* = 0.009, PR *p* = 0.004. Cohort II: ER *p* < 0.001, PR *p* < 0.001), high tumor grade (Cohort I: *p* < 0.001. Cohort II: *p* < 0.001), high expression of the cell proliferation marker Ki-67 (Cohort I: *p* < 0.001. Cohort II: *p* < 0.001) and HER2 positivity (Cohort I: *p* = 0.021) (Table 1).

**Table 1:** Correlation between ANLN expression and clinicopathological characteristics

	Cohort I			Cohort II		
Characteristic	Low ANLN NF n (%)	High ANLN NF n (%)	*p*-value	Low ANLN NF n (%)	High ANLN NF n (%)	*p*-value
Age (years)						
≤ median	42 (67.7)	20 (32.3)		138 (68.0)	65 (32.0)	
> median	32 (50.0)	32 (50.0)	**0.043**	110 (71.0)	45 (29.0)	0.544
Tumor size (mm)						
≤ 20	42 (72.4)	16 (27.6)		99 (76.7)	30 (23.3)	
> 20	32 (47.1)	36 (52.9)	**0.004**	149 (65.1)	80 (34.9)	**0.021**
ER status						
Negative	6 (31.6)	13 (68.4)		50 (43.9)	64 (56.1)	
Positive	68 (63.6)	39 (36.4)	**0.009**	191 (81.6)	43 (18.4)	**<0.001**
PR status						
Negative	15 (39.5)	23 (60.5)		46 (40.7)	67 (59.3)	
Positive	59 (67.0)	29 (33.0)	**0.004**	188 (82.5)	40 (17.5)	**<0.001**
Grade (NHG)						
I	16 (100.0)	0 (0.0)		36 (97.3)	1 (2.7)	
II	43 (74.1)	15 (25.9)		130 (86.7)	20 (13.3)	
III	15 (28.8)	37 (71.2)	**<0.001**	76 (47.2)	85 (52.8)	**<0.001**
Nodal status						
Negative	41 (63.1)	24 (36.9)		64 (66.0)	33 (34.0)	
Positive	25 (49.0)	26 (51.0)	0.129	183 (70.4)	77 (29.6)	0.423
Ki-67						
≤ 10%	50 (96.2)	2 (3.8)		101 (91.8)	9 (8.2)	
> 10%	22 (30.6)	50 (69.4)	**<0.001**	142 (59.4)	97 (40.6)	**<0.001**
HER2 status						
0–2+	72 (61.5)	45 (38.5)		193 (70.4)	81 (29.6)	
3+	2 (22.2)	7 (77.8)	**0.021**	28 (58.3)	20 (41.7)	0.095

*ER* estrogen receptor, *PR* progesterone receptor, *NHG* Nottingham histological grade, *HER2* human epidermal growth factor receptor 2

Age was defined as years at diagnosis. Positive ER and PR expression was considered as >10%. Chi square test, linear-by-linear test or Fisher’s exact test were used to test the significance between groups. Significant correlations (*p* < 0.05) are indicated by bold numbers

### High ANLN nuclear fraction is associated with poor patient survival

**Fig. 3:**
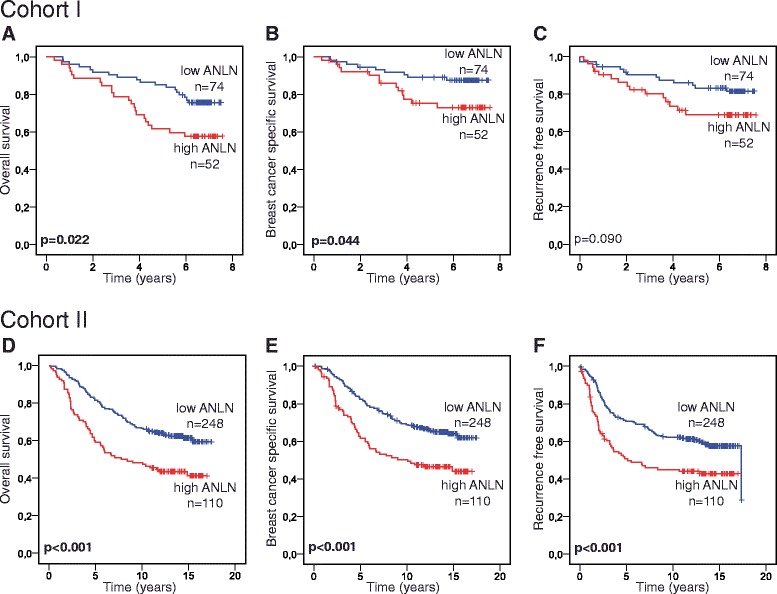
Association of ANLN expression with survival. In cohort I, high ANLN nuclear fraction was significantly associated with a poor outcome in overall survival (**a**) and breast cancer specific survival (**b**) but not in recurrence free survival (**c**). High ANLN nuclear fraction was significantly correlated to a shorter overall survival (**d**), breast cancer specific survival (**e**) and recurrence free survival (**f**) in cohort II

Next, we analyzed the relationship between ANLN expression and prognosis. In cohort I, breast cancer patients with high ANLN NF had a significantly reduced OS (*p* = 0.022) and BCSS (*p* = 0.044) (Fig. 3a, b). Kaplan-Meier analysis and log-rank test for RFS showed a similar trend (Fig. 3c). Similarly, a univariable cox regression model showed that high ANLN NF was significantly associated with a reduced OS (HR = 2.05; 1.10–3.82, 95% CI, *p* = 0.024) and this association remained significant in multivariable analysis (HR = 1.93; 1.03–3.62, 95% CI, *p* = 0.039) when adjusted for age (Table 2). In cohort II, Kaplan-Meier analysis and log-rank test revealed that breast cancer patients with high ANLN NF had a significantly shorter OS (*p* < 0.001), BCSS (*p* < 0.001) and RFS (*p* < 0.001) than patients with a low ANLN NF (Fig. 3d-f). These findings were also confirmed in a univariable cox regression model (OS: HR = 1.91; 1.39–2.63, 95% CI, *p* < 0.001. BCSS: HR = 1.93; 1.38–2.69, 95% CI, *p* < 0.001. RFS: HR = 1.75; 1.27–2.40, 95% CI, *p* = 0.001). ANLN NF was also an independent predictor of OS (HR = 1.61; 1.09–2.39, 95% CI, *p* = 0.018), BCSS (HR = 1.58; 1.05–2.38, 95% CI, *p* = 0.027) and RFS (HR = 1.67; 1.13–2.48, 95% CI, *p* = 0.010), when adjusted for Ki-67, tumor size, hormone receptor status, HER2 status, nodal status and age using multivariable cox regression models (Table 2).

**Table 2:** Cox regression analysis of ANLN NF in relation to OS, BCSS and RFS

	Univariable analysis		Multivariable analysis	
	HR (95% CI)	*p*-value	HR (95% CI)	*p*-value
Cohort I				
OS	2.05 (1.10–3.82)	**0.024**	1.93 (1.03–3.62)	**0.039** ^a^
BCSS	2.34 (1.00–5.47)	0.051		
RFS	1.88 (0.90–3.97)	0.095		
Cohort II				
OS	1.91 (1.39–2.63)	**<0.001**	1.61 (1.09–2.39)	**0.018** ^b^
BCSS	1.93 (1.38–2.69)	**<0.001**	1.58 (1.05–2.38)	**0.027** ^b^
RFS	1.75 (1.27–2.40)	**0.001**	1.67 (1.13–2.48)	**0.010** ^b^

Low ANLN NF(ref) *vs* high ANLN NF

*HR* hazard ratio, *CI* confidence interval, *ref* referent group, *ER* estrogen receptor, *PR* progesterone receptor, *HER2* human epidermal growth factor receptor 2

Significant correlations (*p* < 0.05) are indicated by bold numbers

^a^Multivariable analysis included adjustment for age

^b^Multivariable analysis included adjustment for Ki-67, tumor size, ER, PR, HER2, nodal status and age

### ANLN is not a predictive marker for tamoxifen response

Tamoxifen, a Selective Estrogen Receptor Modulator (SERM), functions as an anti-estrogen [24, 25] and is a widely used adjuvant treatment for patients with early-stage ER positive breast cancer [26, 27]. However, as not all ER positive breast cancer patients respond to tamoxifen treatment [28] and our finding of a strong correlation between ANLN expression and ER status, the possible association between ANLN expression and tamoxifen response was explored. As the patients in cohort II had been included in a randomized prospective tamoxifen trial, we investigated the potential predictive value of ANLN with regard to tamoxifen response in this cohort. The distribution of ANLN NF was similar in the treatment and control arms (Additional file 1: Figure S1). No significant association was seen between ANLN NF and response to tamoxifen treatment, when using Kaplan-Meier analysis and log-rank test (Additional file 2: Figure S2).

### Validation of ANLN protein expression on the transcript level

**Fig. 4:**
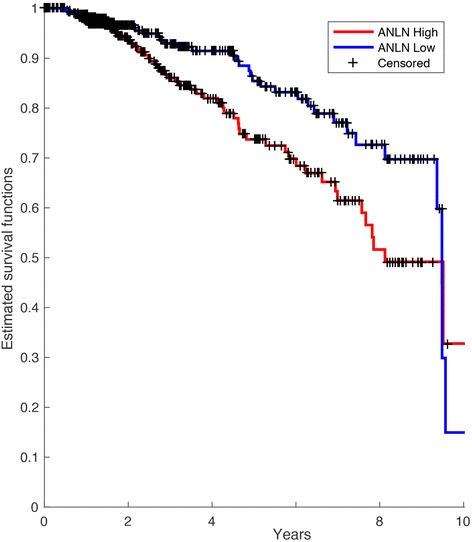
Association of ANLN mRNA expression with survival. Kaplan-Meier survival curve based on gene-expression data and survival information from a publicly available DNA microarray dataset showed that high ANLN expression was significantly associated with poor survival

Analysis of the mRNA sequencing data (RNA-Seq) in the Cancer Genome Atlas showed that breast cancer patients with high expression of ANLN in tumor tissue had a poorer prognosis as compared to patients with low levels of ANLN. A Kaplan-Meier survival estimate, using the median value of ANLN expression as cut-off, showed a significant (*p* = 0.02) difference between patients with tumors expressing high and low levels of ANLN, respectively (Fig. 4). The 5-year survival for the ANLN high group was 74% as compared to 85% for the ANLN low group.

### ANLN depletion leads to cell cycle arrest and altered cell morphology

**Fig. 5:**
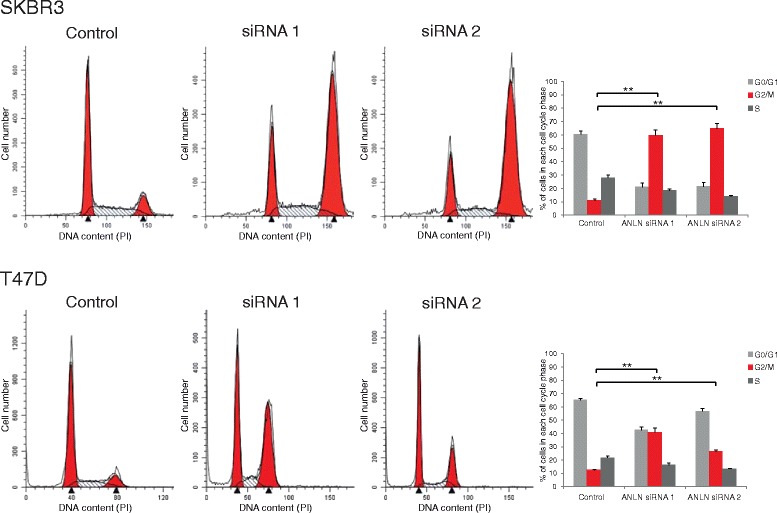
Association of ANLN expression with cell cycle arrest. Flow cytometry-generated data showed that ANLN depletion lead to a significant accumulation of cells in the G2/M phase of the cell cycle 3 days after siRNA knockdown, in both SKBR3 and T47D cells

**Fig. 6:**
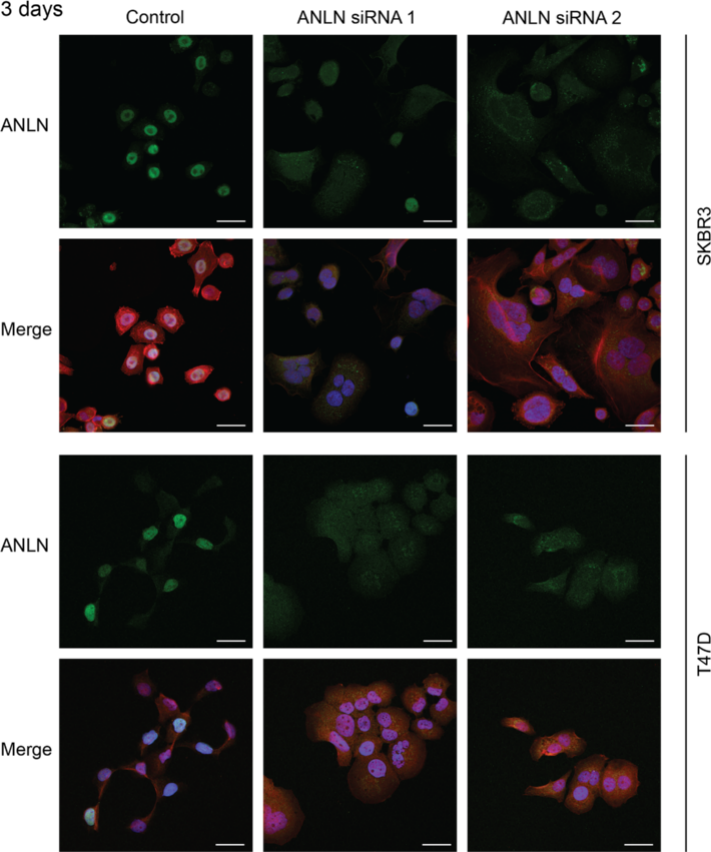
Influence of ANLN on cell morphology. Immunofluorescent staining of SKBR3 and T47D breast cancer cell lines showed efficient knockdown of ANLN nuclear expression by two different siRNAs. ANLN siRNA knockdown induced a larger cell size and cells with multiple nuclei compared to controls in both cell lines examined. ANLN was stained with FITC (*green*), nuclei were stained with DAPI (*blue*) and actin filaments were stained with TRITC (*red*). Scale bars 30 μm

All in vitro experiments were performed at least three times and given results represent a mean value of these experiments. To study the functional relevance of ANLN in breast cancer cells, the expression of ANLN was suppressed in two independent breast cancer cell lines, SKBR3 and T47D, using siRNA-mediated gene knockdown. The distribution of cells in the cell cycle was measured as ANLN has been reported as an important mediator of cell cycle progression. Flow cytometry-generated data showed a significant accumulation of cells in the G2/M phase of the cell cycle 3 days following siRNA transfection in both SKBR3 and T47D cells (Fig. 5). A similar, although not significant, accumulation of cells in the G2/M phase was observed in both cell lines analyzed 5 days after siRNA knockdown of ANLN (Additional file 3: Figure S3). Analysis of cells with reduced expression of ANLN by immunofluorescent staining showed a marked increase in the appearance of large, poly-nucleated cells (Fig. 6 and Additional file 4: Figure S4).

### ANLN depletion induces cellular senescence

**Fig. 7:**
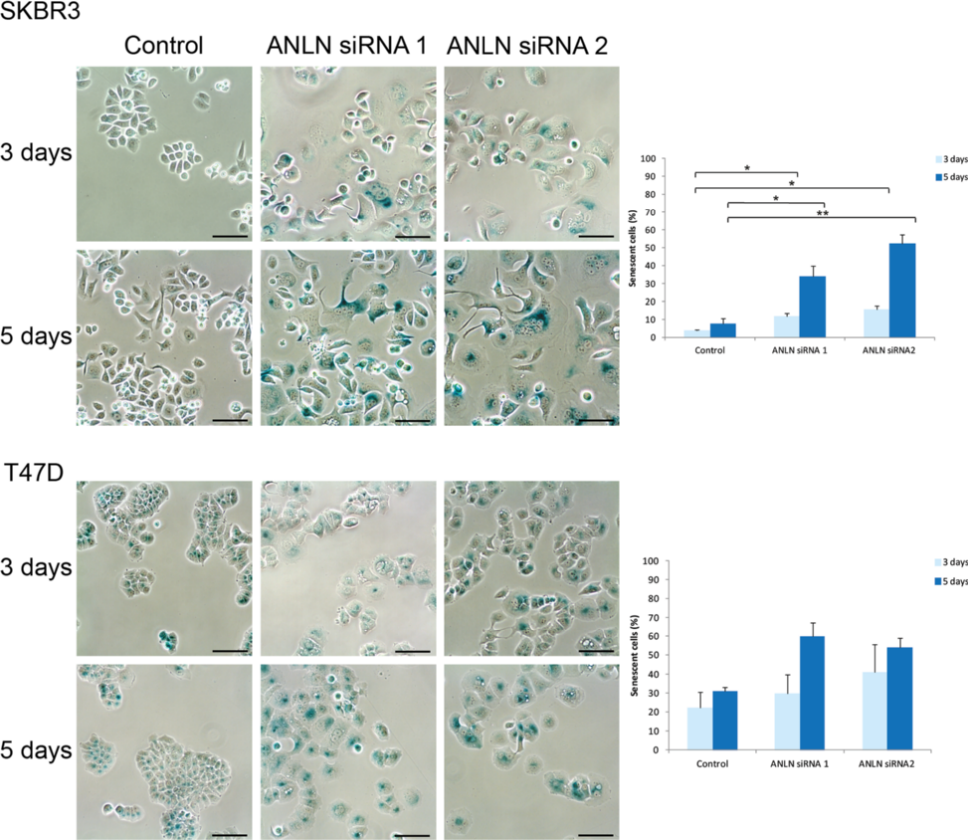
Association of ANLN expression with senescence. Transient knockdown of ANLN expression induced significant levels of cellular senescence compared to control in SKBR3 cells up to 5 days after initiation of ANLN depletion. Similar, but not significant, result was observed for T47D cells. Scale bars 60 μm

As the altered cell morphology was indicative of cellular senescence, this phenotype was further investigated using a β-galactosidase staining assay. The analysis confirmed that the knockdown of ANLN expression induced significant levels of cellular senescence compared to control cells in SKBR3 cells, both 3 and 5 days after initiation of ANLN depletion (Fig. 7 and Additional file 5: Figure S5). Similar findings were observed for T47D cells, although the increase of senescent cells was only significant for ANLN siRNA2 five days after ANLN knockdown.

### ANLN knockdown leads to decreased cyclin expression

**Fig. 8:**
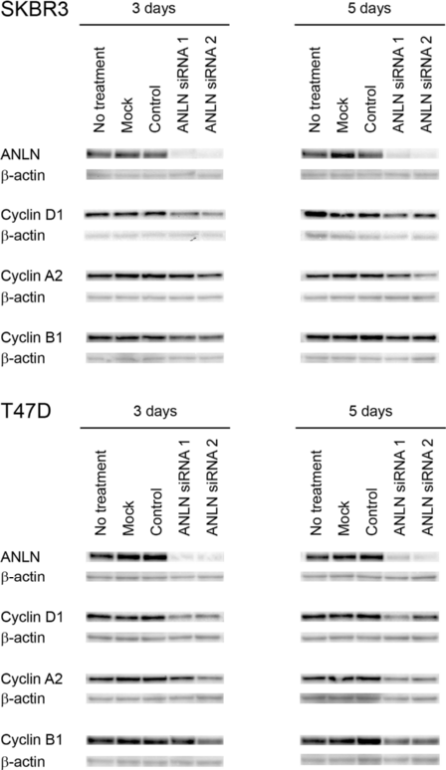
Influence of ANLN on cell cycle associated proteins. Western blot analysis showed efficient knockdown of ANLN expression by two different siRNAs. Overall, ANLN siRNA knockdown resulted in lower expression of cyclin D1, cyclin A2 and cyclin B1compared to controls in SKBR3 and T47D breast cancer cell lines. B-actin was used as a loading control

A potential correlation between ANLN expression and the cell cycle associated proteins cyclin D1, A2 and B1 was investigated to further study the role of ANLN in the cell cycle. The dynamics and levels of decreased cyclin expression varied between cell lines and time points (Fig. 8). Using Spearman’s correlation test on IHC data from cohort II we found that ANLN NI was significantly correlated to cyclin D1 expression (Cyclin D1 NF: correlation coefficient = 0.130, *p* = 0.015. Cyclin D1 NI: correlation coefficient = 0.142, *p* = 0.008). Three days after inducing a transient knockdown of ANLN in SKBR3 cells, a reduced western blot signal for cyclin D1 was noted. In T47D cells, a weaker signal for cyclin D1 was seen up to 5 days after knockdown. ANLN siRNA knockdown also resulted in a reduction of cyclin A2 in both cell lines, which was maintained up to 5 days, although this was most evident in T47D cells. For cyclin B1, ANLN siRNA reduced the western blot signal in T47D cells up to 5 days after knockdown while the signal reduction in SKBR3 cells was more evident after 3 days.

## Discussion

The identification of novel prognostic and predictive factors is crucial for the development of personalized medicine for cancer patients. The present study aimed at further examining the prognostic value and functional role of ANLN in breast cancer. O’Leary et al [13] showed that a high ANLN intensity was associated with poor prognosis in breast cancer patients. The IHC based ANLN expression data in the present study is well consistent with these findings, however, our data are based on manual assessment of the fraction of positive tumor cells which appears superior and a more robust, reproducible and convenient assessment as compared with estimating IHC intensities.

Two independent breast cancer patient cohorts were used to investigate the potential impact of ANLN expression on survival. In both TMA cohorts, a high nuclear fraction of ANLN was significantly associated with a poor OS when adjusted for well-known clinicopathological factors, using multivariable cox regression models. Furthermore, in the larger cohort II, multivariable cox regression analysis showed that a high nuclear fraction of ANLN was significantly associated with a reduced BCSS and RFS independent of the cell proliferation marker Ki-67, tumor size, hormone receptor status, HER2 status, nodal status and age. However, in cohort I, ANLN nuclear fraction was not significantly associated with BCSS nor RFS, which may be due to the low number of events for BCSS and RFS in this comparatively small patient cohort. Our results based on immunohistochemistry and ANLN protein expression are consistent with findings on the transcript level. Based on quantitative RNA-Seq data available from the Cancer Genome Atlas we showed that high levels of ANLN mRNA in breast cancer tissue were associated with shorter survival of patients as compared to patients with low levels of ANLN mRNA. These findings are also in agreement with a previous study on breast cancer patients showing ANLN as a gene associated with increased risk for recurrence of breast cancer [29]. Overall, our cohort data and the results from transcriptomics analyses support the finding that ANLN is a strong independent prognostic biomarker for breast cancer.

IHC combined with tissue microarrays containing well-characterized tumors provide an attractive strategy for both discovery and validation of new cancer biomarkers [30]. Despite the obvious need for improved patient stratification and numerous studies suggesting novel cancer biomarkers, clinical decision making is at large dependent on morphological assessment and IHC staining based on proliferation (Ki-67), hormone receptor status and expression of HER2 [31]. The expression pattern of the ANLN protein, based on several validated antibodies also including the antibody used in the present study, is presented in the Human Protein Atlas (www.proteinatlas.org) in a multitude of human normal and cancer tissues. The Human Protein Atlas provides gene expression data on both the mRNA and protein level, including underlying IHC-based images, for a vast majority of all human protein coding genes [22, 32, 33]. Our results show that IHC-based ANLN expression provides a distinct and reproducible staining pattern, suggesting a role as supplement to the prognostic breast cancer biomarkers currently used in the clinic.

Previous studies have shown that the ANLN protein is involved in cytokinesis, more specifically in the formation of the cleavage furrow during late anaphase and telophase [3, 34, 35]. Similar to the recently published findings by Zhou et al [14], we found that transient knockdown of ANLN protein expression in breast cancer cell lines resulted in a significant accumulation of cells in the G2/M phase of the cell cycle. Although experiments performed on only two different cell lines with differences in ER expression do not allow for general conclusions, we found that the impact of ANLN on these two breast cancer cells were similar, independent of ER status. Similar to Zhou et al we detected decreased expression of cyclin D1 in response to ANLN depletion. However, in contrast to Zhou et al, we also observed that transient ANLN knockdown reduced levels of cyclin B1, which is needed for G2/M transition, and Cyclin A2, involved in S/G2 transition. The shift in cell cycle distribution was most likely caused by an arrest of cells in G2/M phase, with cells being unable to complete mitosis, resulting in large and multi-nucleated (mainly double-nucleated) cells. This is consistent with a previous study by Oegema et al [3], who observed slower cell cleavage leading to furrow regression and multi-nucleated cells in response to anti-ANLN antibody microinjection. In addition, Straight et al [35] also observed multi-nucleated HeLa cells following treatment with ANLN siRNA. The cell cycle findings also correlated well with the increased number of senescent cells that we observed.

The result from the present study validates previous findings of high nuclear expression of ANLN being associated with a more aggressive breast cancer phenotype. It is noteworthy that even high grade breast cancer with severe nuclear atypia can be void of ANLN expression and that ANLN is a prognostic factor independent of proliferation (Ki-67 expression), suggesting possible additional roles for ANLN not directly linked to cytokinesis. Although there was a trend showing a possible treatment predictive value of ANLN for tamoxifen treatment, the correlation between ANLN expression and response to tamoxifen was not significant. Nevertheless, breast cancer patients with a high tumor-specific ANLN expression may benefit from more aggressive treatment due to the apparent “poor prognosis” phenotype associated with high ANLN expression.

## Conclusions

In conclusion, our data shows that ANLN expression is a strong prognostic factor in breast cancer and essential for cell cycle progression. We also show that depletion of ANLN induces cellular senescence in breast cancer cell lines. Further prospective studies are needed to establish a potential role in the clinical management of breast cancer patients with tumors showing a high level of ANLN expression.

## Additional files

Additional file 1: Figure S1. Distribution of ANLN nuclear fraction with regard to tamoxifen treatment. Distribution of ANLN nuclear fraction was analyzed with regard to tamoxifen treatment in cohort II, a randomized prospective tamoxifen trial. The distribution of ANLN nuclear fraction was similar in the treatment and control arms with the majority of tumors expressing less than 25% of ANLN nuclear staining. (PDF 25 kb)
Additional file 2: Figure S2. Association of ANLN expression with tamoxifen response. The potential treatment predictive value of ANLN with regard to tamoxifen response was investigated in cohort II, a randomized prospective tamoxifen trial. ANLN nuclear fraction was not a significant predictor of tamoxifen response as shown by overall survival (A, D) breast cancer specific survival (B, E) or recurrence free survival (C, F). (PDF 50 kb)
Additional file 3: Figure S3. A. Association of ANLN expression with cell cycle arrest in SKBR3 cells. Flow cytometry-generated data showed that ANLN depletion lead to a significant accumulation of cells in the G2/M phase of the cell cycle 3 days after siRNA knockdown in SKBR3 cells. This accumulation was clearly visible but not statistically significant 5 days after ANLN siRNA knockdown. B. Association of ANLN expression with cell cycle arrest in T47D cells. Flow cytometry-generated data showed that ANLN depletion lead to a significant accumulation of cells in the G2/M phase of the cell cycle 3 days after siRNA knockdown in T47D cells. This accumulation was clearly visible but not statistically significant 5 days after ANLN siRNA knockdown. (ZIP 146 kb)
Additional file 4: Figure S4. A. Influence of ANLN on cell morphology in SKBR3 cells. Immunofluorescent staining of SKBR3 breast cancer cell lines showed that ANLN siRNA knockdown induced a larger cell size and cells with multiple nuclei compared to controls up to 5 days after knockdown. ANLN was stained with FITC (green), nuclei were stained with DAPI (blue) and actin filaments were stained with TRITC (red). Scale bars 30 μm. B. Influence of ANLN on cell morphology in T47D cells. Immunofluorescent staining of T47D breast cancer cell lines showed that ANLN siRNA knockdown induced a larger cell size and cells with multiple nuclei compared to controls up to 5 days after knockdown. ANLN was stained with FITC (green), nuclei were stained with DAPI (blue) and actin filaments were stained with TRITC (red). Scale bars 30 μm. (ZIP 4784 kb)
Additional file 5: Figure S5. Association of ANLN expression with senescence. Transient knockdown of ANLN expression induced significant levels of cellular senescence compared to controls in SKBR3 cells up to 5 days after initiation of ANLN depletion. Significant levels of cellular senescence compared to controls were noted in T47D cells 5 days after ANLN siRNA knockdown. Similar, but not significant, result was observed 3 days after knockdown. Scale bars 60 μm. (TIF 14662 kb)
