# Supplementary figures and images for: ANLN is a prognostic biomarker independent of Ki-67 and essential for cell cycle progression in primary breast cancer
Source: BMC Cancer. 2016 Nov 18;16:904. doi: 10.1186/s12885-016-2923-8 (PMC5116155; doi:10.1186/s12885-016-2923-8)

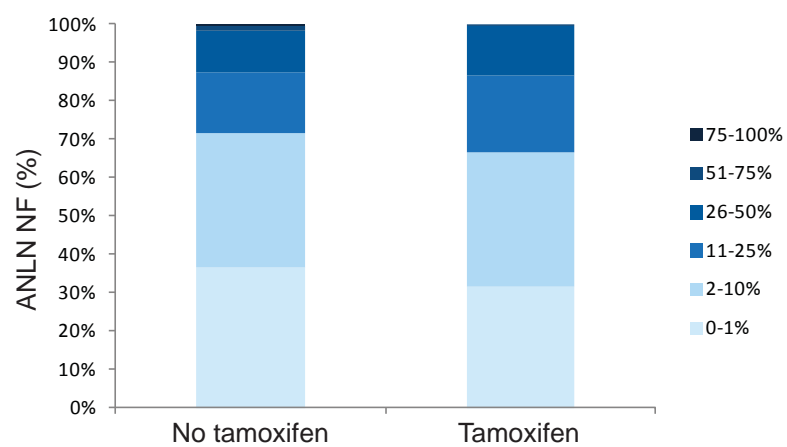

Supplement: Additional file 1: Figure S1. — Distribution of ANLN nuclear fraction with regard to tamoxifen treatment. Distribution of ANLN nuclear fraction was analyzed with regard to tamoxifen treatment in cohort II, a randomized prospective tamoxifen trial. The distribution of ANLN nuclear fraction was similar in the treatment and control arms with the majority of tumors expressing less than 25% of ANLN nuclear staining. (PDF 25 kb) [file 12885_2016_2923_MOESM1_ESM.pdf]

## High ANLN

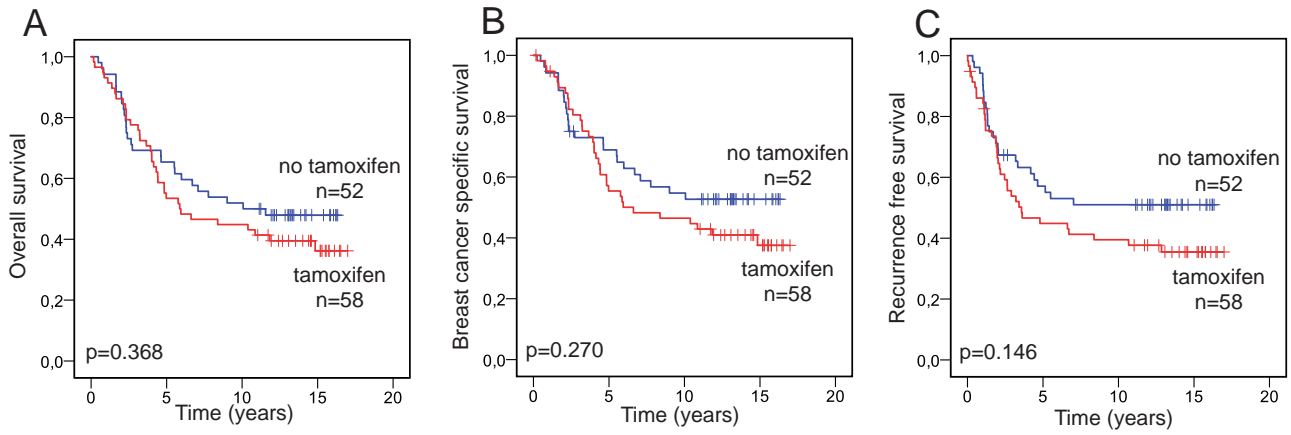

## Low ANLN

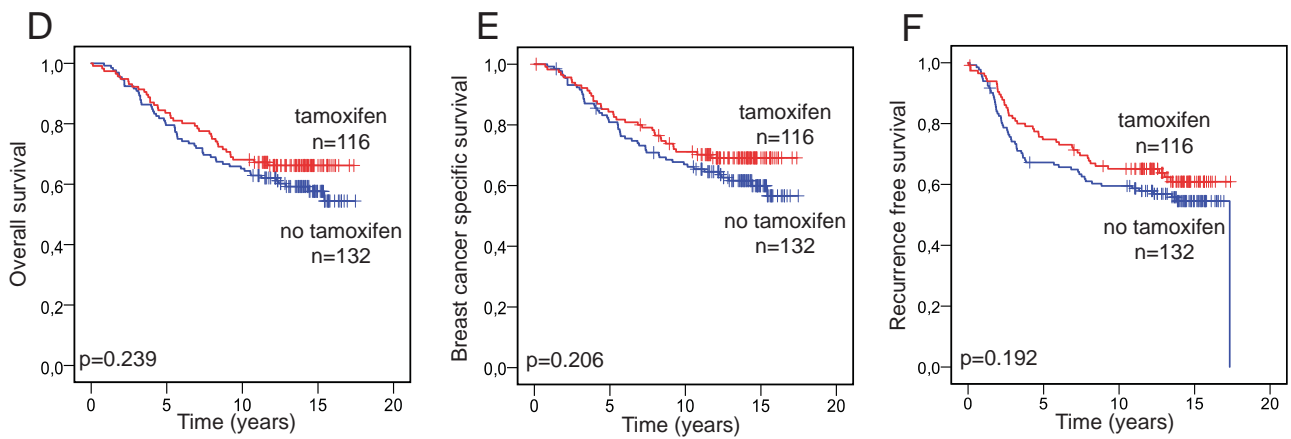

Supplement: Additional file 2: Figure S2. — Association of ANLN expression with tamoxifen response. The potential treatment predictive value of ANLN with regard to tamoxifen response was investigated in cohort II, a randomized prospective tamoxifen trial. ANLN nuclear fraction was not a significant predictor of tamoxifen response as shown by overall survival (A, D) breast cancer specific survival (B, E) or recurrence free survival (C, F). (PDF 50 kb) [file 12885_2016_2923_MOESM2_ESM.pdf]

SKBR3

3 days

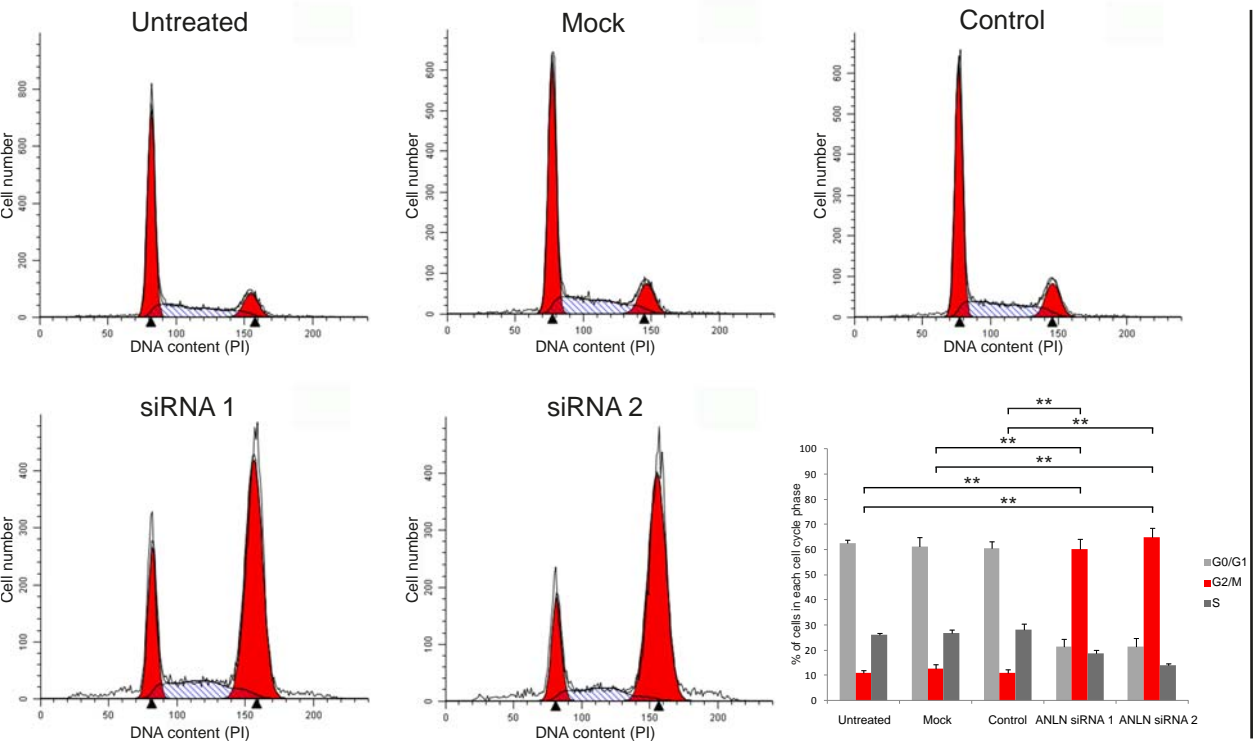

5 days

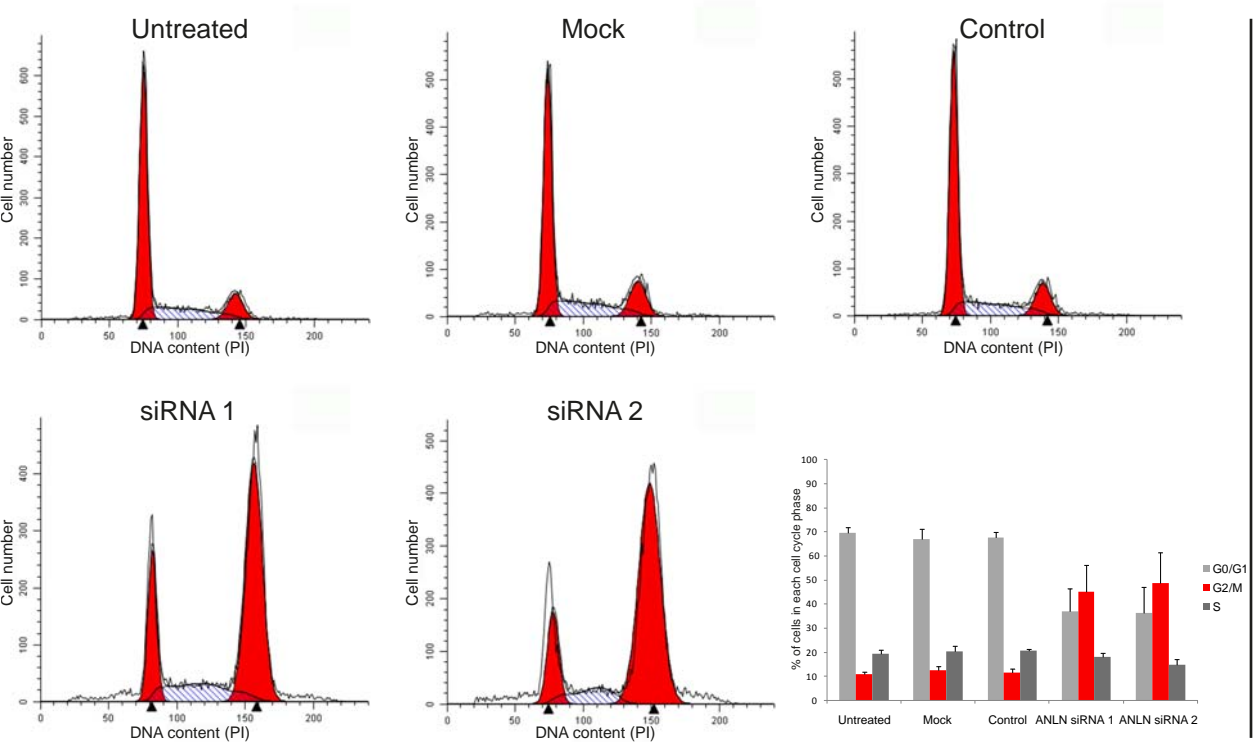

Supplement: Additional file 3: Figure S3. — A. Association of ANLN expression with cell cycle arrest in SKBR3 cells. Flow cytometry-generated data showed that ANLN depletion lead to a significant accumulation of cells in the G2/M phase of the cell cycle 3 days after siRNA knockdown in SKBR3 cells. This accumulation was clearly visible but not statistically significant 5 days after ANLN siRNA knockdown. B. Association of ANLN expression with cell cycle arrest in T47D cells. Flow cytometry-generated data showed that ANLN depletion lead to a significant accumulation of cells in the G2/M phase of the cell cycle 3 days after siRNA knockdown in T47D cells. This accumulation was clearly visible but not statistically significant 5 days after ANLN siRNA knockdown. (ZIP 146 kb) [file 12885_2016_2923_MOESM3_ESM.zip › Supplementary Figure 3A - Association of ANLN expression with cell cycle arrest in SKBR3 cellsR3.pdf]

T47D

3 days

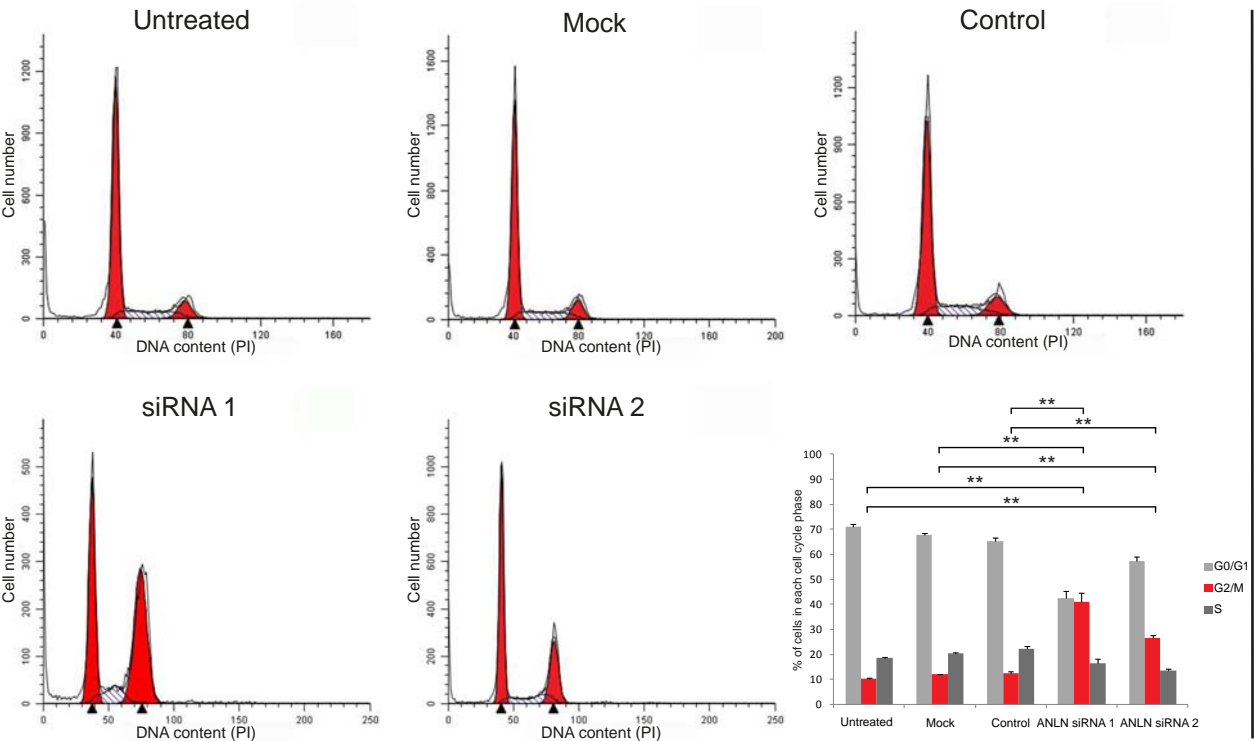

5 days

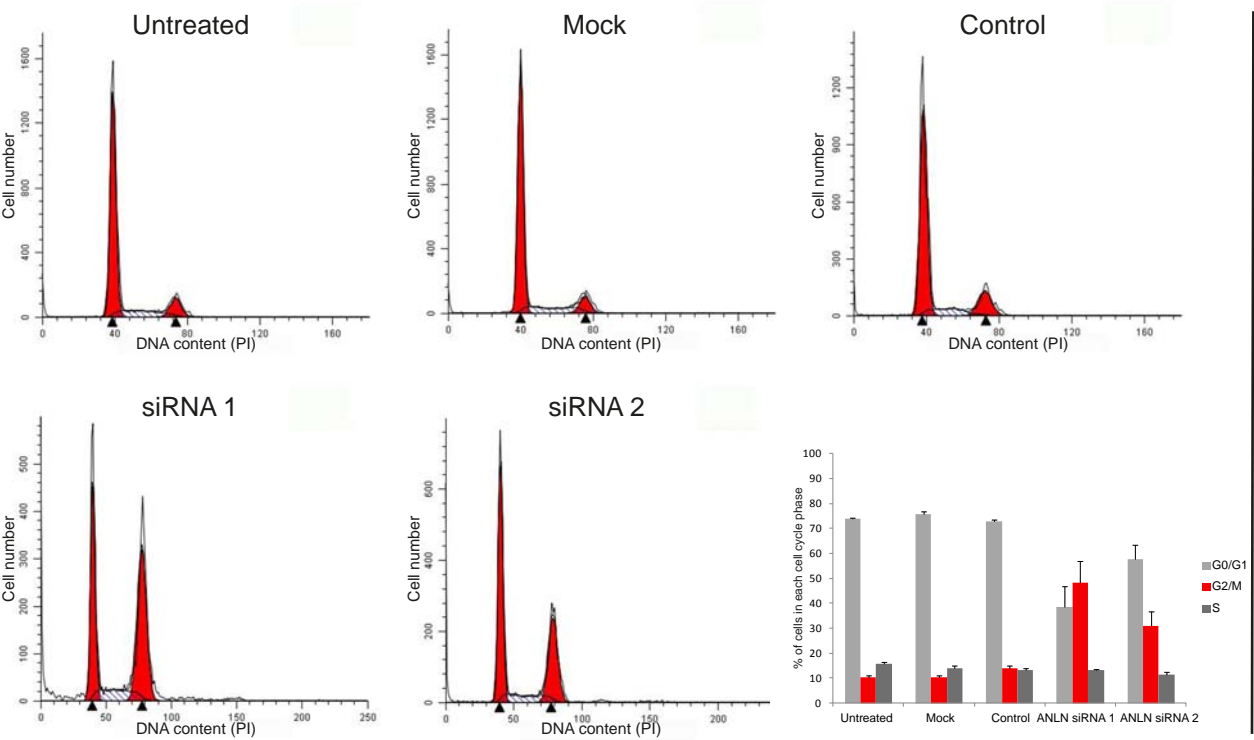

Supplement: Additional file 3: Figure S3. — A. Association of ANLN expression with cell cycle arrest in SKBR3 cells. Flow cytometry-generated data showed that ANLN depletion lead to a significant accumulation of cells in the G2/M phase of the cell cycle 3 days after siRNA knockdown in SKBR3 cells. This accumulation was clearly visible but not statistically significant 5 days after ANLN siRNA knockdown. B. Association of ANLN expression with cell cycle arrest in T47D cells. Flow cytometry-generated data showed that ANLN depletion lead to a significant accumulation of cells in the G2/M phase of the cell cycle 3 days after siRNA knockdown in T47D cells. This accumulation was clearly visible but not statistically significant 5 days after ANLN siRNA knockdown. (ZIP 146 kb) [file 12885_2016_2923_MOESM3_ESM.zip › Supplementary Figure 3B - Association of ANLN expression with cell cycle arrest in T47D cellsR3.pdf]

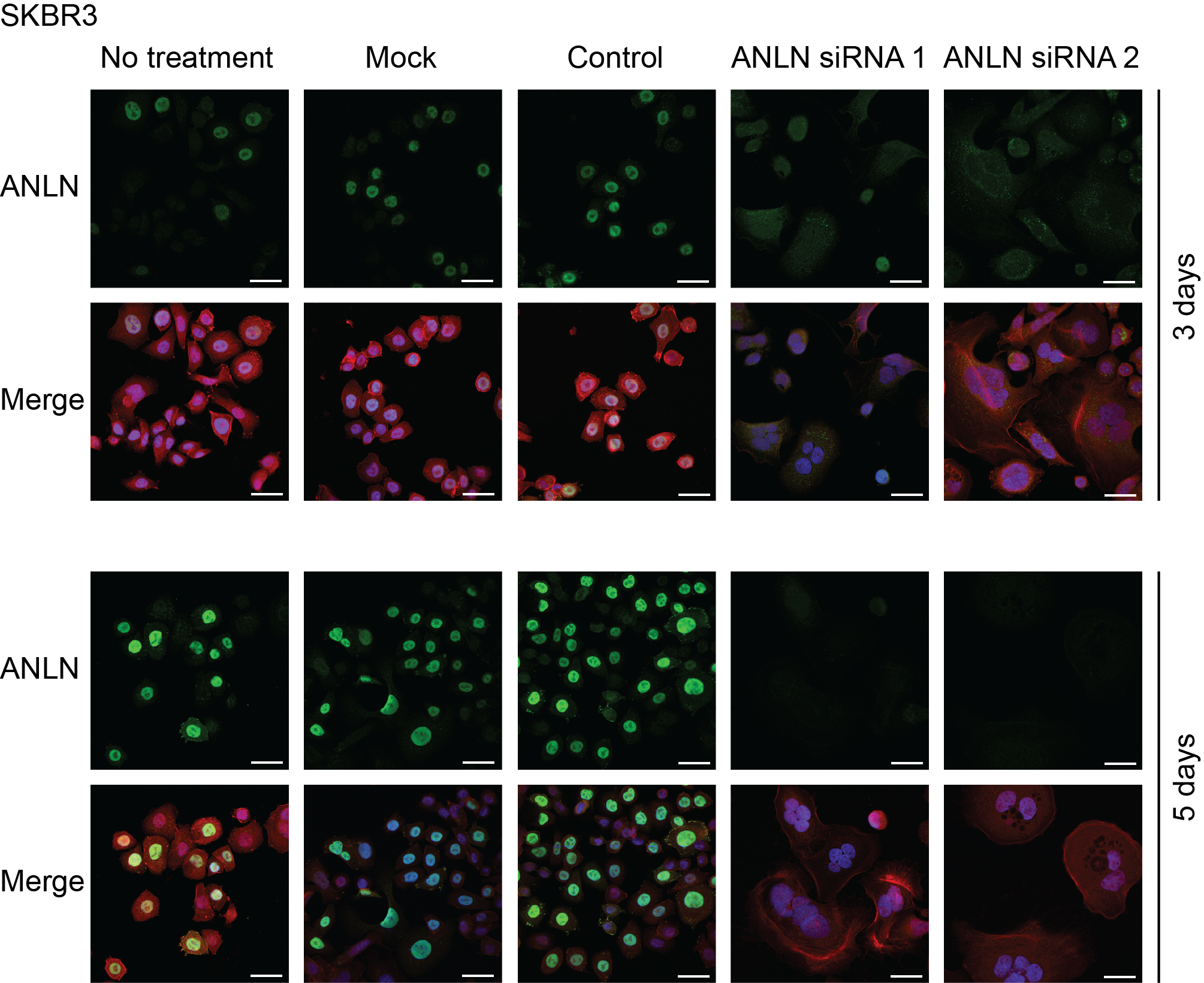

Supplement: Additional file 4: Figure S4. — A. Influence of ANLN on cell morphology in SKBR3 cells. Immunofluorescent staining of SKBR3 breast cancer cell lines showed that ANLN siRNA knockdown induced a larger cell size and cells with multiple nuclei compared to controls up to 5 days after knockdown. ANLN was stained with FITC (green), nuclei were stained with DAPI (blue) and actin filaments were stained with TRITC (red). Scale bars 30 μm. B. Influence of ANLN on cell morphology in T47D cells. Immunofluorescent staining of T47D breast cancer cell lines showed that ANLN siRNA knockdown induced a larger cell size and cells with multiple nuclei compared to controls up to 5 days after knockdown. ANLN was stained with FITC (green), nuclei were stained with DAPI (blue) and actin filaments were stained with TRITC (red). Scale bars 30 μm. (ZIP 4784 kb) [file 12885_2016_2923_MOESM4_ESM.zip › Supplementary Figure 4A_scalebarsR3_30.tif]

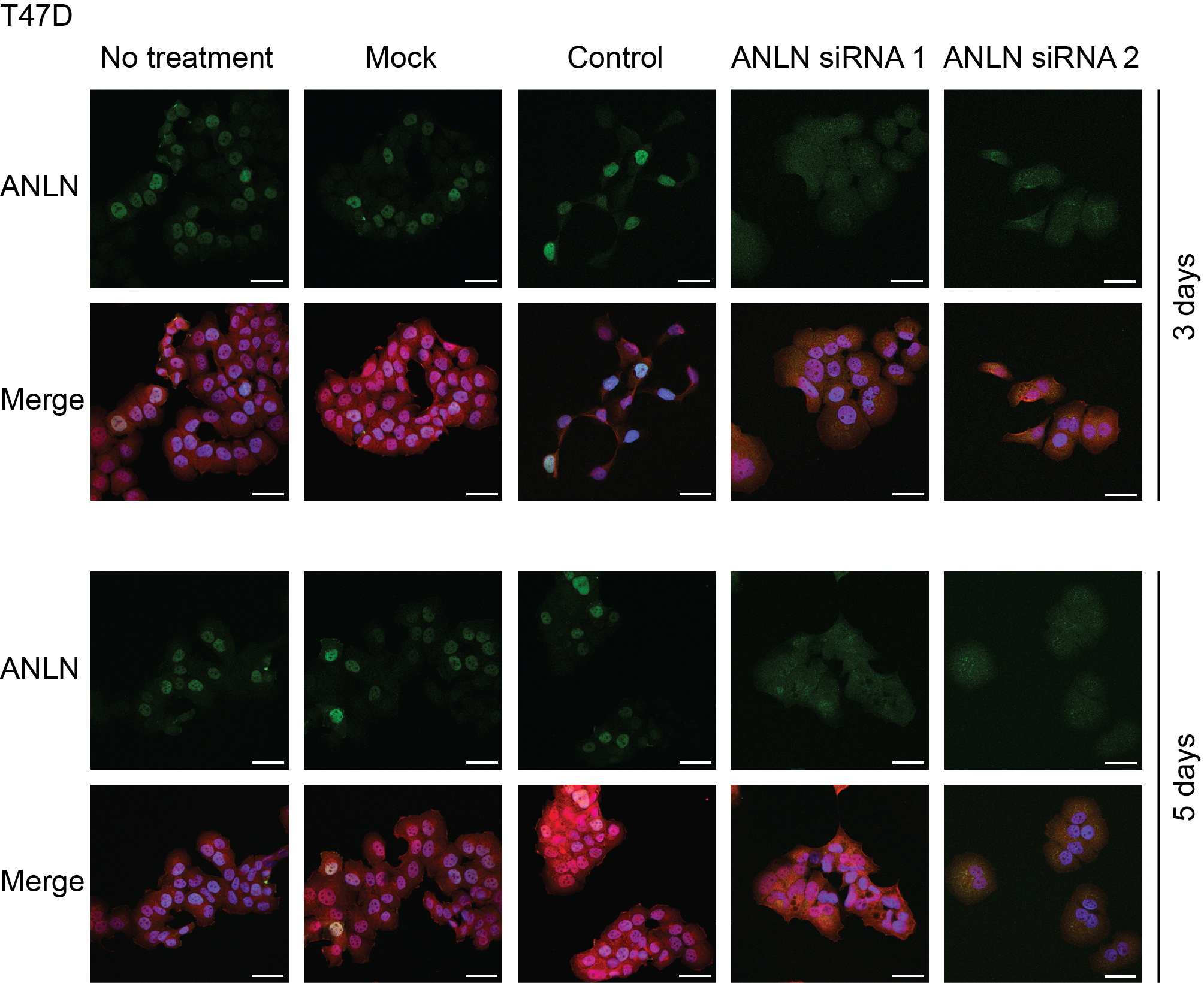

Supplement: Additional file 4: Figure S4. — A. Influence of ANLN on cell morphology in SKBR3 cells. Immunofluorescent staining of SKBR3 breast cancer cell lines showed that ANLN siRNA knockdown induced a larger cell size and cells with multiple nuclei compared to controls up to 5 days after knockdown. ANLN was stained with FITC (green), nuclei were stained with DAPI (blue) and actin filaments were stained with TRITC (red). Scale bars 30 μm. B. Influence of ANLN on cell morphology in T47D cells. Immunofluorescent staining of T47D breast cancer cell lines showed that ANLN siRNA knockdown induced a larger cell size and cells with multiple nuclei compared to controls up to 5 days after knockdown. ANLN was stained with FITC (green), nuclei were stained with DAPI (blue) and actin filaments were stained with TRITC (red). Scale bars 30 μm. (ZIP 4784 kb) [file 12885_2016_2923_MOESM4_ESM.zip › Supplementary Figure 4BscalebarsR3_30.tif]

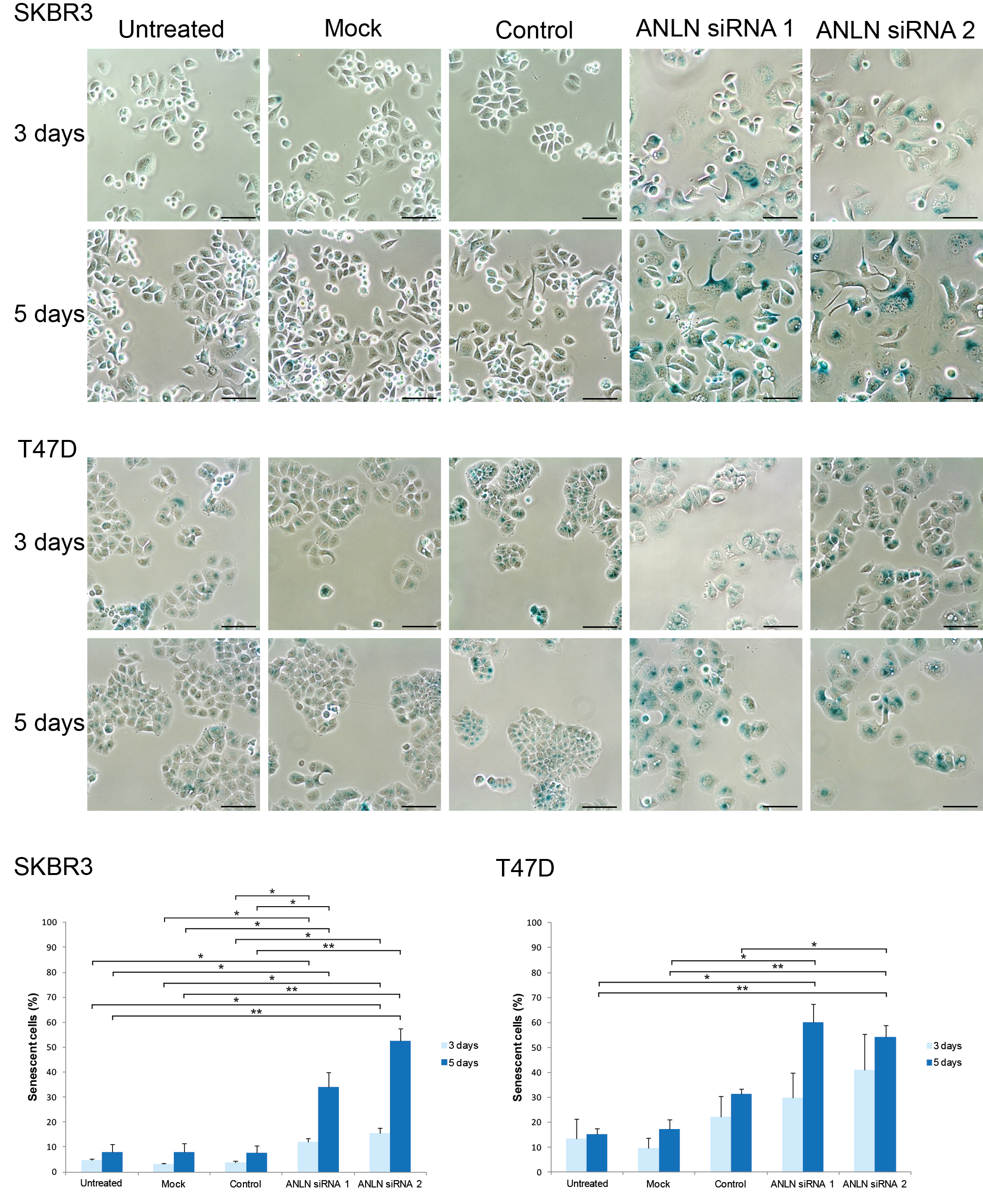

Supplement: Additional file 5: Figure S5. — Association of ANLN expression with senescence. Transient knockdown of ANLN expression induced significant levels of cellular senescence compared to controls in SKBR3 cells up to 5 days after initiation of ANLN depletion. Significant levels of cellular senescence compared to controls were noted in T47D cells 5 days after ANLN siRNA knockdown. Similar, but not significant, result was observed 3 days after knockdown. Scale bars 60 μm. (TIF 14662 kb) [file 12885_2016_2923_MOESM5_ESM.tif]
